# Influence of Land Mosaic Composition and Structure on Patchy Populations: The Case of the Water Vole (*Arvicola sapidus*) in Mediterranean Farmland

**DOI:** 10.1371/journal.pone.0069976

**Published:** 2013-07-16

**Authors:** Ricardo Pita, António Mira, Pedro Beja

**Affiliations:** 1 Centro de Investigação em Biodiversidade e Recursos Genéticos, Universidade de Évora, Évora, Portugal; 2 EDP Biodiversity Chair, Centro de Investigação em Biodiversidade e Recursos Genéticos, Universidade do Porto, Campus Agrário de Vairão, Vairão, Portugal; The Australian National University, Australia

## Abstract

The ability of patchy populations to persist in human-dominated landscapes is often assessed using focal patch approaches, in which the local occurrence or abundance of a species is related to the properties of individual patches and the surrounding landscape context. However, useful additional insights could probably be gained through broader, mosaic-level approaches, whereby whole land mosaics with contrasting patch-network and matrix characteristics are the units of investigation. In this study we addressed this issue, analysing how the southern water vole (*Arvicola sapidus*) responds to variables describing patch-network and matrix properties within replicated Mediterranean farmland mosaics, across a gradient of agricultural intensification. Patch-network characteristics had a dominant effect, with the total amount of habitat positively influencing both the occurrence of water voles and the proportion of area occupied in land mosaics. The proportions of patches and area occupied by the species were positively influenced by mean patch size, and negatively so by patch isolation. Matrix effects were weak, although there was a tendency for a higher proportion of occupied patches in more intensive, irrigated agricultural landscapes, particularly during the dry season. In terms of conservation, results suggest that water voles may be able to cope well with, or even be favoured by, the on-going expansion of irrigated agriculture in Mediterranean dry-lands, provided that a number of patches of wet herbaceous vegetation are maintained within the farmland mosaic. Overall, our study suggests that the mosaic-level approach may provide a useful framework to understand the responses of patchy populations to land use change.

## Introduction

Understanding the impacts of land use change on biodiversity is a core issue in landscape ecology and conservation biology [1], [2]. Such impacts have often been assessed by sampling species occupancy or local abundance at replicated sites or habitat patches, and then testing the influence of sets of variables describing the patches and the landscape structure within a defined area surrounding the sampling units [3]. This ‘focal-patch’ approach has provided valuable information on key factors determining species persistence in fragmented landscapes, contributing decisively to guide conservation management efforts [4], [5]. Recently, however, it has become apparent that additional insights could be gained by scaling up the focus of attention to mosaics of multiple land uses, based on the idea that ‘whole’ landscapes may have emergent properties that are not fully captured when concentrating only on individual habitat patches [6]–[7]. Mosaic-level inference requires replicated sampling of both response variables and predictors at the land mosaic scale, and generally interpret variation in species distribution or abundance across land mosaics in relation the extent of habitat, composition of the mosaic, and spatial configuration of elements [6]–[7]. This approach has been considered particularly useful for informing conservation management, because it enhances the matching between the scale of research and the scale at which human-dominated landscapes are typically managed [8].

The mosaic-level approach has proved useful for drawing inferences on generalist and wide ranging species that occupy multiple land uses and elements within land mosaics (e.g., [9]–[11]). Much less is known about the utility of this approach for specialised species that are restricted to discrete habitat patches and have limited movement capability, thereby occurring as metapopulations, or, in a broader sense, as patchy populations [12], [13]. These species have successfully been studied from the focal patch perspective, which produced a large body of evidence showing that patch occupancy and local abundance are mostly related to patch size, quality and geographical isolation [14]–[16], and the permeability of the surrounding matrix to the movement of individuals [17]–[18]. However, metapopulation theory suggests that patch and matrix characteristics should also have effects at the level of patch networks besides those at the level of individual patches, which in turn may have far-reaching consequences for species persistence in fragmented landscapes [14]. Although these ideas have mostly been explored through analytic and simulation models (e.g., [19], [20]), they have also been addressed by a few empirical field studies showing that network occupancy (i.e., the presence/absence of the species in each network) and the proportion of patches occupied, may be influenced by network level features such as total habitat area, total number of patches, average patch size and overall connectivity among patches [21]–[24]. These studies used patch networks rather than individual patches as the units of replication, thus sharing clear similarities with the mosaic level approach. However, the taxonomic scope of these studies has been limited and they have not considered the effects of the matrix surrounding patch networks, thus calling for further empirical evidence on the value of mosaic level approaches for understanding the responses of patchy populations to land use change [14], [25].

Here we provide a case study on the use of mosaic-level inference to understand the effects of agricultural change on patchy populations. We focused on a rodent species of conservation concern, the southern water vole (*Arvicola sapidus*), analysing the responses of its patchy populations to variation in patch-network and matrix features in Mediterranean farmland. Specifically, we sampled replicated land mosaics selected across a gradient of agricultural intensification, estimating for each land mosaic (i) the presence/absence of water voles, and the proportions of (ii) patches and (iii) area occupied by the species. In line with previous network-level metapopulation studies [21]–[24] and the land mosaic paradigm [6], [7], we hypothesised that these population characteristics could be largely explained by relatively coarse mosaic properties such as the extent of habitat available, the number, size, shape and spatial configuration of habitat patches, and the composition of the surrounding matrix. Results of the study were then used to discuss the potential of mosaic-level approaches to produce general management guidelines for the conservation of patchy populations in human-dominated landscapes.

## Methods

### Ethics Statement

The Portuguese nature conservation authority (ICNB - Instituto da Conservação da Natureza e Biodiversidade) authorized the sampling of southern water voles, including the capture and handling of animals (Licences 30/2006/CAPT, 156/2006/CAPT, 312/2007/CAPT, 50/2008/CAPT). The results described in this paper were based on the detection of field signs (mainly droppings and runways on vegetation), without any type of direct contact with voles (e.g., capture, handling, manipulation, transportation, or captivity) at any stage of this study, and so it did not require approval by an Institutional Animal Care and Use Committee (IACUC) or equivalent animal ethics committee. Surveys were carried out in private properties, where authorization of access was obtained. The southern water vole is not a protected species under EU legislation or under the Portuguese national legislation.

### Study area and species

**Figure 1:**
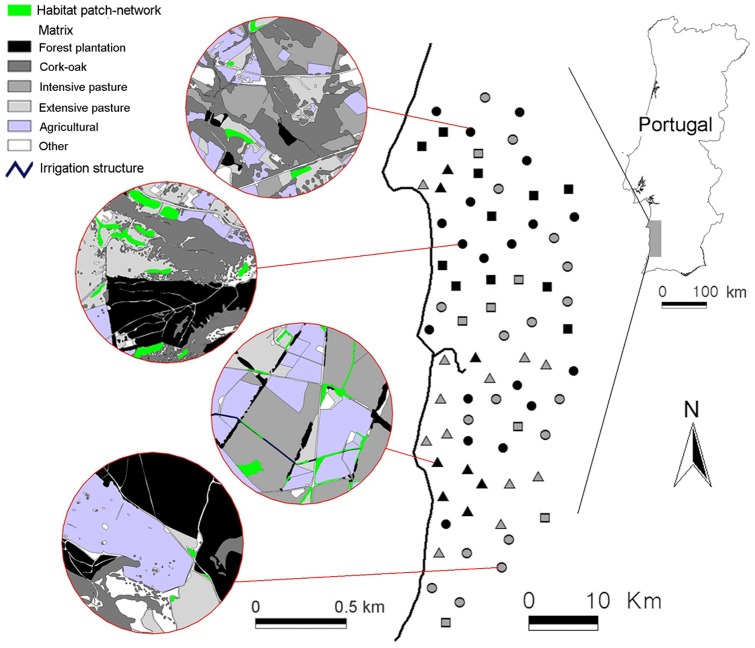
Location of the study region and sampling sites (land mosaics) used to investigate water vole occupancy according to patch-network and matrix characteristics. Examples of four land mosaics with different patch-network and matrix characteristics are also presented. Triangles, circles and squares represent sampling sites surveyed respectively in 2006 (n = 20), 2007 (n = 37), and 2008 (n = 18). Colours indicate the sampling season of surveys: dry season (black, n = 38) and wet season (grey, n = 37) (see text for details).

The study was conducted in an area of about 2000 km^2^ in south-west Portugal (37° 21′−38° 04′ N, 08° 51′−08° 30′ W, Fig. 1). The climate is Mediterranean with oceanic influence. Mean monthly temperatures (i.e., sum of daily means/number of days in the month) range between 10°C (January) and 22°C (August), and average annual rainfall is around 650 mm, of which >80% falls in October to March (wet season) [11], [26]. The region is characterized by agricultural mosaics of different land uses dominated by crop and livestock production, which together cover over 65% of the study area [11]. Wood cover is restricted to a few woodlots and hedges with planted pines and eucalyptus delimiting irrigated fields, while semi-natural habitats occur marginally in dunes, entrenched stream valleys, and cork oak woodlands surrounding the farmed areas [11], [26]. Surface waters are mostly associated with small intermittent streams and temporary ponds that flood during the rainy season and dry out in summer, whereas permanent water bodies are scarce and mostly associated with irrigation infrastructures [27]. Despite the overall trend for agricultural intensification since the early 1990s [28], some areas have been abandoned or maintain extensive agriculture, resulting in many landscape types and ecological gradients that reflect different management options across the region, which are likely to affect water vole populations.

Water voles in the study area are restricted to scattered patches of tall (≈>30 cm) and dense (ca. 100% cover) wet herbaceous vegetation dominated by grasses, sedges, rushes, and reeds, which often occupy gently sloping and soft bank-margins of ponds, small streams and irrigation ditches [27], [29]. Although throughout most of its range the water vole occurs close to permanent and stable water bodies (e.g., [30]–[32]), in dry Mediterranean landscapes such as our study area, individuals often persist in seasonally flooded habitats that may become dry during the hottest months [27], [29], [33]–[35]. Within habitat patches, water voles form discrete and easily recognized breeding colonies [27], [33], with individuals typically showing strong site fidelity to their home ranges (mean size of about 900 m^2^, [27]) and usually moving less then about 30 m between successive days [34]. Mean lifespan of individuals is around 3–5 months [34], and mating may occur within polygynous or monogamous systems, depending on local habitat quality and population density [27], [34], [36]. As in other regions (e.g., [33]–[35]), suitable habitat patches in the study area are often separated from each other by hundreds of meters of inhospitable matrix, and thus breeding colonies are likely to support distinct demographic units connected by dispersal (mean distances of about 600–800 m [34], [35]). In addition, there is evidence for local extinction and colonization events, which suggests a metapopulation-like dynamics [33]–[35], similarly to the congeneric *A. amphibius* populations in many regions from Northern Europe (e.g., [38]–[40]).

### Sampling design

The study was based on 75 land mosaics (sampling units) encompassing a wide range of patch network and matrix characteristics, and reflecting the regional gradient of agricultural intensification (Fig. 1). Land mosaics corresponded to circles of 500 m radius (78.5 ha), which were randomly selected across the study area, constrained to a minimum distance between its centres of 2 km (mean±se [range] nearest neighbour distance between centres = 3.6±0.07 km [2.5–5.8 km]). The size of sampling units was considered adequate to describe farmland mosaics, given the range of daily movements usually made by individual water voles [27], [34]. In addition, this size was large enough to contain several habitat patches suitable for water voles, while it was sufficiently small to allow replication across the region.

Within each land mosaic, we surveyed the distribution of water voles, and characterised mosaic structure and composition. Because water vole surveys and habitat mapping were time consuming, and we wanted to maximize sampling size at the expense of temporal replication within sites [41], each land mosaic was sampled only once, either in the wet (October-March) or the dry (April-September) season, and in just one of three sampling years. Specifically, land mosaic surveys were conducted in 2006 (20 land mosaics), 2007 (37), and 2008 (18), encompassing the dry (38 land mosaics) and the wet seasons (37) (Fig.1).

### Land mosaic structure and composition

In each land mosaic, we characterised the network of patches available to water voles using eight variables reflecting the overall quantity of habitat, and the average size, shape and connectivity of patches (Table 1). Habitat patches were identified during systematic field surveys, and mapped through GPS recordings made along their borders. Patch suitability was judged irrespective of water vole presence, based on habitat preferences derived from previous radiotracking studies [27], [29]. We considered as a habitat patch any discrete area covered by suitable wet herbaceous vegetation, which was separated from other patches by >5 m of unsuitable habitat. This small distance was used because artificial gaps such as paved roads are likely to function as effective barriers in the context of the short daily movements of individuals within their home-ranges [34], [42]. The minimum area considered for patch delimitation was 50 m^2^, which was well below the minimum home range size recorded for water voles in the study area (ca. 200 m^2^ [27]). Habitat mapping data was incorporated in a vector-based Geographic Information System (GIS, ArcView 3.2, Redlands, CA, 1999), and variables characterising the patch network were extracted using the Spatial Analyst software [43].

**Table 1:** Summary statistics of habitat-network and matrix variables recorded per land mosaic, and overall and seasonal occupancy patterns of water voles in south-western Portugal.

Set/variable (units)	N^a^	Mean± se	Range	Transformation
Patch Network Characteristics				
Number of suitable habitat patches	75	5.5±0.5	0–17	logarithmic
Total habitat area (ha)	75	1.9±0.3	0–12.9	logarithmic
Mean patch size (ha)	69	0.4±0.1	0.3–2.2	logarithmic
Mean patch perimeter-area ratio (m/m^2^)	69	0.2±0.01	0.1–0.7	logarithmic
Area weighted mean fractal dimension	69	1.5±0.01	1.3–1.9	-
Mean distance among patches (m)	69	363.8±29.9	22.1–1000	logarithmic
Mean distance nearest patch (m)	69	194.7±35.5	5.7–1000	logarithmic
Total patch edge density (km/km^2^)	75	2.7±0.3	0–12.3	logarithmic
Matrix Characteristics				
Forest plantations (%)	75	16.7±2.6	0–93.6	angular
Agricultural (%)	75	12.9±2.2	0–84.1	angular
Intensive pastures (%)	75	16.0±2.7	0–81.6	angular
Extensive pastures (%)	75	20.8±2.4	0–76.1	angular
Cork oak (%)	75	22.1±2.8	0–56.0	angular
Irrigation structures (km/km^2^)	75	0.3±0.1	0–4.8	logarithmic
Water vole variables				
Land mosaic occupancy (0/1)	75	0.59	0–1	–
*Dry season*	38	0.55	0–1	–
*Wet season*	37	0.62	0–1	–
Patch occupancy rate (%)	69	35.0±3.5	0–100	logarithmic
*Dry season*	33	33.3±5.3	0–100	logarithmic
*Wet season*	36	36.5±5.9	0–100	logarithmic
Extent of occupancy (%)	44	1.4±0.04	0.07–10.4	logarithmic
*Dry season*	21	1.0±0.03	0.15–2.4	logarithmic
*Wet season*	23	1.8±0.1	0.07–10.4	logarithmic

a Sample size (N) is not constant, because some variables could only be computed for a subset of the land mosaics studied, and because different mosaics were sampled in the wet and the dry seasons.

We also characterised the matrix surrounding patch networks, using six variables reflecting the dominant land uses and the density of irrigation structures (irrigation channels and drainage ditches) (Table 1). The latter variable was used as an index of agriculture intensification. Land use and irrigation structure mapping was based on recent (2005) high resolution (0.5 m/pixel) aerial photographs, and ground validation. Five main land use classes (i.e. those representing at least 10% of overall cover) were considered: forest plantations (pines and eucalyptus planted for wood production or for crop protection from maritime winds); agricultural land (land used for the production of cereals, vegetables and other crops); improved pastures (sown and irrigated pastures intended for cattle grazing); extensive pastures (natural pastures and fallows lightly grazed by cattle); and cork oak, *Quercus suber*, woodlands. Land use mapping was done at the scale of 1:1000, although finer scales (down to 1:10) were sometimes used for editing and correcting digitizing errors. To reduce eventual subjectivity in polygon delineation, the minimum polygon area admitted for land use mapping was 5 m^2^.

### Vole surveys

The presence and area effectively occupied by water voles within suitable habitat patches were estimated from systematic field searches for their typical presence signs, including fresh latrines or scattered droppings, runways, burrows, and green grass clippings [32]–[34], [44]. Water vole signs are easily recognized in the field [44] and provide a reliable basis for large scale surveys (e.g., [32], [33]). For instance, a single visit of up to 20 minutes was considered adequate to accurately evaluate the presence of water voles in pond-like habitat patches larger than 1 ha [33]. However, because false absences can strongly affect the results of habitat modelling [41], we minimised imperfect detection by increasing sampling effort and surveying carefully the entire surface of each habitat-patch, rather than stopping searches at first detection. Surveys were always carried out in periods without precipitation, both during the sampling period and at least the previous two days, to avoid eventual disappearance of presence signs [44]. Based on past experience from extensive trapping and radiotracking of water voles in the study area, we are confident that this sampling strategy was adequate for the purposes of this study. Mapping of the areas effectively occupied by voles was made by collecting a set of GPS point locations delimiting their boundaries, which were then incorporated in the GIS. Within habitat patches, presence signs distanced by>30 m (aproximately the mean diameter of a hypothetical circular home-range) were mapped as distinct vole areas.

Surveys were used to compute three variables reflecting mosaic-level properties of water vole populations in the study area (Table 1): (i) land mosaic occupancy ( = network occupancy), i.e. the presence/absence of water voles in each land mosaic; (ii) patch occupancy rate, i.e. the proportion of patches occupied by water voles in relation to the total number of patches recorded in each land mosaic; and (iii) extent of occupancy, i.e. the proportion of area effectively occupied by voles relative to the total area of each land mosaic. Land mosaic occupancy was selected because it was considered suitable in previous empirical studies to estimate the attributes of habitat networks influencing metapopulation persistence [22]–[24]. Patch occupancy rate was used because it is a network level property that is expected to be affected by the average characteristics of individual patches [14], though this has seldom been tested (but see, e.g., [21]). Extent of occupancy was used as a coarse index of overall population size (i.e., number of breeding water voles) within each land mosaic, based on previous radiotracking studies showing that adult water voles have well-defined home ranges, which vary little across seasons in size and overlap between neighbouring individuals [27].

### Data analysis

Statistical analysis aimed to identify relationships between variables reflecting water vole distribution and abundance, and variables reflecting the structure and composition of patch networks and the surrounding matrix. We conducted analysis considering both the entire dataset and the dataset divided per wet and dry seasons, because previous studies pointed out possible seasonal variation of habitat use by water voles at the patch scale [29], [33], suggesting that mosaic-level effects could also vary across seasons.

Prior to statistical analysis, all variables except indexes and binary descriptors were transformed to approach normality and to reduce the influence of extreme values, using the angular transformation for proportional data and the logarithmic transformation for other continuous variables (Table 1). Analysis were based on axis extracted from Principal Component Analysis (PCA) with Varimax rotation, in order to reduce dimensionality, solve multicollinearity problems, and identifying the main ecological gradients (eigenvalues >1). Separate PCAs were performed for sets of variables describing (i) patch-networks and (ii) the surrounding matrix, because we wanted to identify potential relationships between the two sets (see below), and because we wanted to assess the relative influence of each set on water voles. The patch-network PCA included only the subset of 69 land mosaics where suitable water vole habitats were found (Table 1). For land mosaics including only one patch (n = 6), estimates of mean distance among patches and mean distance to nearest patch were set at 1000 m, which is higher than the maximum estimates for land mosaics with >1 patches (i.e. 714 m, n = 63). All PCAs were implemented using the open source software R 2.14.2 [45] and the package “psych” [46].

To assess whether patch-network gradients were influenced by matrix gradients, we used the subset of 69 land mosaics to perform a Redundancy Analysis (RDA, [47]), followed by Multiscale Ordination (MSO) integrating the geographical coordinates of sampling units [48], [49]. The MSO is a geostatistical tool based on the general assumption that autocorrelated residuals can alter the results of statistical tests, and consists in performing a constrained ordination on the RDA to check the resultant spatial structure [48], [50]. This method allows the partitioning of results into distance classes, the distinction between induced spatial dependence and spatial autocorrelation, and the use of variograms to check the spatial variance profile of the canonical and residual ordination axis [48]. MSO was performed using 1000 Monte Carlo permutation tests, and considered an interval width of 5 km (about the maximum nearest distance among land mosaic centroids), which resulted in 9 distance classes (from 5 to 75 km). In RDA, the significance of relationships was calculated by performing 1000 permutations [51]. These analyses were conducted in R using the package “vegan” [52].

Generalized linear mixed-effect models (GLMM) were used to analyse the relationships between explanatory and response variables, under an information-theoretic model selection and averaging approach. The season and year of surveys were included as random effects, thereby accounting for potential lack of independence between land mosaics sampled in the same time periods [53]. Water vole occurrence in land mosaics was modelled with binomial error distribution and logit link, considering the subset of 69 land mosaics with suitable water vole habitats. Patch occupancy rates and extent of occupancy were modelled with Gaussian error distribution and identity link. Patch occupancy was analysed using the subset of 69 land mosaics including suitable habitat patches, while extent of occupancy was modelled using the 44 land mosaics where water voles were recorded (see Table 1 and Results). All GLMMs were fitted by maximum likelihood estimation using the function lmer in the R package “lme4” [54].

Model selection involved a two-stage procedure based on the Akaike Information Criterion adjusted to small samples (AICc), which measures the relative support of fitted models [55]. We started by performing univariate GLMMs to evaluate alternative response curves (linear and quadratic) describing the relationships between each response variable and each set of explanatory variables. For each response variable and ecological gradient, the best fitting curve was carried forward to subsequent analysis, using Akaike weights (wi) as the model selection criteria [55]. In each case, scatterplots and regression diagnostics were used to inspect the shape of the fitted curves and to check for problems resulting from the presence of outliers and influential points [51]. Model AICc were estimated using the R package “MuMIn” [56].

We then developed multivariate GLMMs relating each response variable with patch-network and matrix network gradients. Autocovariate terms (ATC) for spatially correlated responses, as assessed from global Moran's *I* coefficients with associated z-values>1.96 [57], were generated using the inverse distance weighting in the R package “spdep” [58], and then considered in model building. In each case, the best fitting model was selected from all possible main-effect (terms order 1) combinations using the Information Theoretical Approach (ITA) of Burnham and Anderson [55] based on AICc and Akaike weights (wi). Uncertainty in model selection was accounted for using AICc-based multimodel inference and averaging (MI), which uses an estimated weighted average across all models based on model weights [55]. The MI approach provides robust estimates of model parameters and higher accuracy of predictions regarding the magnitude of the effects of ecological gradients on response variables [57], [59]. Unconditional standard errors of estimates were used to evaluate the precision of model average estimates using a 95% confidence interval. Estimates whose confidence limits included zero were viewed as having equivocal meaning [55]. The strength of both patch-network and matrix effects on seasonal occupancy patterns of water voles was further assessed by repeating the MI procedure separately for each season, including the surveyed month in the random-effects part of the models. All ITA-AICc analyses were implemented using the R package “glmulti” [60].

## Results

### Water vole distribution

Water vole habitats were found in 69 out of 75 land mosaics. Overall, there were 413 habitat patches occupying 2.5% of the total area surveyed. About 90% of patches were smaller than 0.8 ha, and about 90% were at less than 250 m from the nearest patch (see Table 1 for detailed summary statistics). Water voles were found at 44 land mosaics, totalling 158 patches occupied, which together covered 0.8% of the landscapes surveyed. Occupancy rates tended to be smaller in the dry season (Table 1), although differences were not significant (t-tests, *p*>0.05). The global Moran's *I* index suggested a positive spatial autocorrelation in water vole land mosaic occupancy (*I* = 0.04, *z-*value>1.96), and a random spatial pattern (*z-*values <|1.96|) for both patch occupancy rate (*I* = 0.02) and extent of occupancy (*I* = −0.01).

### Patch-network and matrix properties of land mosaics

The first three components extracted from the patch-network PCA accounted for 88.2% of the variance in the data (Table 2). The first component (H1) reflected primarily the habitat amount, as indicated by the concurrent increase in the number, total area, and edge density of habitat patches, together with a decrease in the distances to the nearest patch (Table 2). The second component (H2) was primarily related to patch size and shape, as it was positively correlated with the mean size and total area of habitat patches, and negatively correlated with patch perimeter-area ratios and area weighted patch fractal dimensions (Table 2). The third component (H3) was related to habitat isolation, as it was positively correlated with the distances among patches (Table 2). The PCA regarding the landscape matrix extracted three components, together accounting for 77.5% of the variance in the original data (Table 3). The first component (M1) reflected a gradient of irrigated agriculture, as it was positively correlated with the amount of agricultural land and irrigation structures, and negatively so with cork-oak dominated areas. The second component (M2) was interpreted as a gradient reflecting grassland management intensification. The third component (M3) described an increase in forest plantations cover (Table 3).

**Table 2:** Summary results of a principal component analysis based on variables describing the characteristics of habitat patch-networks of water voles in southwestern Portugal (N = 69).

Variable (codes)	Increase in habitat availability (H1)	Increase in patch size (H2)	Increase in patch isolation (H3)
Number of suitable habitat patches	0.93	0.04	−0.07
Total habitat area (ha)	0.79	0.58	−0.11
Mean patch size (ha)	0.34	0.79	−0.06
Mean patch perimeter-area ratio (m/m^2^)	−0.05	−0.95	0.05
Area weighted mean fractal dimension	−0.09	−0.86	0.12
Mean distance among patches (m)	0.09	−0.14	0.97
Mean distance nearest patch (m)	−0.57	−0.05	0.78
Total patch edge density (km/km^2^)	0.95	0.21	−0.05
			
Initial Eigenvalues	4.06	1.65	1.35
% of Variance	50.69	20.60	16.86

Total variance explained 88.2%. Rotation Method: Varimax with Kaiser Normalization. Values in bold indicate |factor loadings| >0.50.

**Table 3:** Summary results of a principal component analysis based on matrix variables characterising the land mosaics surveyed for water voles in southwestern Portugal (N = 75).

Variables (codes)	Increase in irrigated agriculture (M1)	Increase in pasture intensification (M2)	Increase in planted forest (M3)
Forest plantation (%)	−0.01	0.003	0.98
Agricultural (%)	0.79	−0.14	−0.04
Intensive pastures (%)	0.28	0.82	−0.25
Extensive pastures (%)	0.16	−0.82	−0.23
Cork oak (%)	−0.79	−0.11	−0.46
Irrigation structures (km/km^2^)	0.77	0.18	−0.17
			
Initial Eigenvalues	2.01	1.34	1.30
% of Variance	33.55	22.29	21.69

Total variance explained 77.5%. Rotation Method: Varimax with Kaiser Normalization. Values in bold indicate |factor loadings| >0.50.

**Figure 2:**
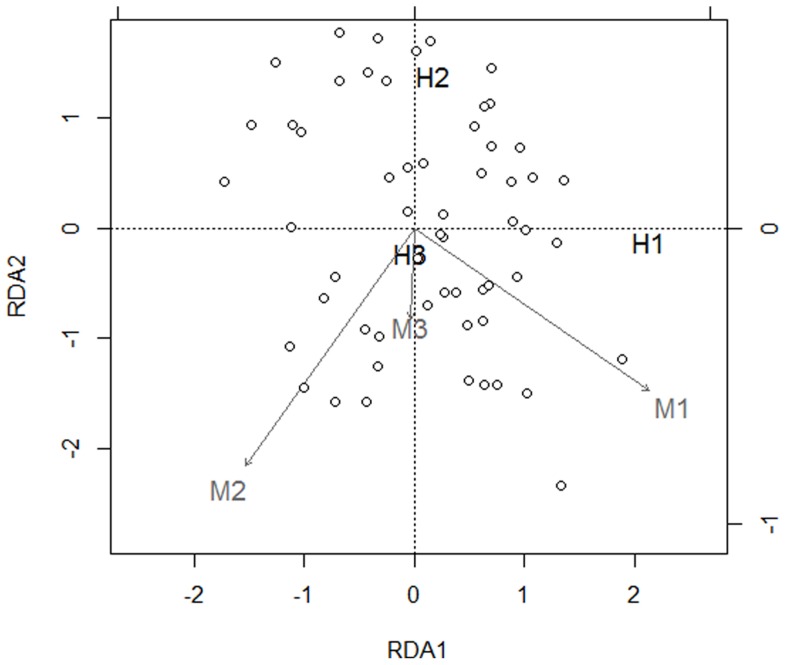
Redundancy Analysis (RDA) relating patch-network and matrix gradients performed for the 69 land mosaics including suitable habitat for water voles. Bi-plot of the first two canonical axes of patch-network (H1, H2, H3) and matrix gradients (M1, M2, M3). Patch-network variables and sites were scaled symmetrically by the square root of eigenvalues. Eigeinvalues for axis 1 = 0.304, and axis 2 = 0.059. Habitat-matrix correlations for the first two axes were 0.996 and 0.942. Explained variation was 0.37, pseudo*-F* = 3.01, *p* = 0.01. Effects of matrix characteristics on patch-network structure were significant in respect to irrigated agriculture (M1, *p*<0.01) and pasture intensification (M2, *p* = 0.02), but not significant regarding forest plantation (M3, *p* = 0.827).

Results of RDA (Fig. 2) suggested that there was a positive relation between irrigated agriculture (M1) and the amount of water vole habitat (H1), along with some tendency for smaller patch sizes (H2). In addition, land mosaics with a higher proportion of intensively managed grasslands (M2) had in general less habitat amount (H1) and smaller patches (H2) than land mosaics including extensive pastures. Integration of spatial information into the RDA by MSO revealed no significant spatial correlation among distance classes (see Fig. S1), suggesting that patch-network characteristics may be explained by matrix gradients, independently of the geographical location of land mosaics within the study region.

### Water vole responses to land mosaic characteristics

Preliminary univariate screening provided strong support for both patch-network and matrix characteristics influencing water vole occupancy patterns within land mosaics (Table 4, Fig. S2). Globally, habitat amount (H1), mean patch size (H2), and irrigated agriculture (M1) had positive effects on water voles. Isolation among habitat patches (H3) showed inconsistent effects, with some support for curvilinear responses in the case of land mosaic occupancy and patch occupancy rate (Table 4, Fig. S2). There was also some support for negative effects of pasture intensification (M2), particularly regarding water voles extent of occupancy within land mosaics, while forest plantation cover (M3) showed weak and generally inconsistent effects on all measures considered (Table 4, Fig. S2).

**Table 4:** Akaike weights (wi) of univariate models fitted to test alternative water vole response curves (linear or quadratic) to the main mosaic gradients describing the habitat-network and the matrix.

		Land mosaic occupancy (n = 69)			Patch occupancy rate (n = 69)			Extent of occupancy (n = 44)		
Fixed effects		Null model	Linear model	Quadratic model	Null model	Linear model	Quadratic model	Null model	Linear model	Quadratic model
Habitat	H1	0.000	0.758 (+)	0.241	0.011	0.734 (+)	0.255	0.002	0.744 (+)	0.254
	H2	0.135	0.505 (+)	0.360	0.068	0.588 (+)	0.344	0.029	0.771 (+)	0.201
	H3	0.030	0.076	0.894 (∩)	0.008	0.296	0.696 (∩)	0.661	0.229 (+)	0.110
Matrix	M1	0.002	0.702 (+)	0.296	0.002	0.765 (+)	0.233	0.237	0.435 (+)	0.328
	M2	0.471	0.401 (−)	0.127	0.568	0.326 (−)	0.107	0.256	0.581 (−)	0.163
	M3	0.541	0.202	0.256 (∩)	0.454	0.183	0.363 (∩)	0.557	0.237 (−)	0.204

Comparisons included the null model (i.e. fitted only to the random component). The directions of associations between land mosaic occupancy measures and predictors are presented for response curves used in multivariate analysis: (+) positive, (−) negative, (∩) unimodal (see Fig. S2).

In multivariate modelling, there was a very strong effect of habitat amount (H1) on land mosaic occupancy (Table 5), which was particularly strong during the wet season (Table 6). There was also strong support for the positive effects of mean patch size (H2) and irrigated agriculture (M1) on patch occupancy rates, and a negative effect of patch isolation (H3) (Table 5), which were particularly strong during the dry season (Table 6). The extent of occupancy was only influenced by patch-network characteristics, with positive effects of habitat amount (H1) and mean patch size (H2) (Table 5). These effects were particularly supported during the wet season, when an additional negative effect of patch isolation (H3) was also evident (Table 6).

**Table 5:** Summary results of information-theoretic model selection and multimodel inference on the relationships between mosaic occupancy of water voles across spatial resolutions and the mosaic gradients describing habitat-networks (H1, H2, H3) and matrix types (M1, M2, M3), and the autocovariate terms (ATC) for spatially correlated responses (see text).

Response	Wi (best model)	Model averaging			
		Predictor	# Models	Selection probability	Estimate (Unconditional 95%CI)
Land mosaic occupancy	0.1363	H1	256	0.9763	0.192 (0.071, 0.313)
		H2	256	0.7809	0.089 (−0.030, 0.209)
		H3	256	0.8087	−0.093 (−0.212, 0.025)
		H3^2^	256	0.2558	0.000 (−0.021, 0.022)
		M1	256	0.7357	0.086 (−0.046, 0.218)
		M2	256	0.2158	−0.000 (−0.023, 0.023)
		M3	256	0.2146	−0.002 (−0.031, 0.027)
		M3^2^	256	0.2915	−0.014 (−0.068, 0.040)
		ATC	256	0.2694	0.117 (−0.407, 0.642)
Patch occupancy rate	0.2321	H1	128	0.6718	0.071 (−0.057, 0.200)
		H2	128	0.9624	0.141 (0.045, 0.238)
		H3	128	0.9931	−0.175 (−0.269, −0.082)
		H3^2^	128	0.2701	−0.004 (−0.031, 0.022)
		M1	128	0.9868	0.175 (0.072, 0.278)
		M2	128	0.2176	0.001 (−0.022, 0.025)
		M3	128	0.2104	−0.001 (−0.027, 0.025)
		M3^2^	128	0.2660	−0.010 (−0.054, 0.033
Extent of occupancy	0.3629	H1	32	1.0000	0.380 (0.276, 0.484)
		H2	32	1.0000	0.378 (0.287, 0.470)
		H3	32	0.7800	−0.091 (−0.214, 0.031)
		M1	32	0.8193	0.082 (−0.016, 0.179)
		M2	32	0.2994	0.016 (−0.040, 0.071)
		M3	32	0.1825	0.003 (−0.022, 0.029)

The table provides Akaike weights of the best fitting models (wi) for each response variable, the number of models including each predictor, the selection probabilities, and model averaged regression coefficient with 95% confidence intervals. Predictors included in the best models are underlined. Coefficient estimates whose 95%CI excluded 0 are in bold.

**Table 6:** Summary results of information-theoretic model selection and multimodel inference performed separately for each season to compare seasonal relationships between mosaic occupancy of water voles across spatial resolutions, and the mosaic gradients describing habitat-networks (H1, H2, H3) and matrix types (M1, M2, M3), and the autocovariate terms (ATC) for spatially correlated responses (see text).

	Dry season					Wet season				
Response	Wi (best model)	Model averaging				Wi (best model)	Model averaging			
		Predictor	# Models	Selection probability	Estimate (Unconditional 95%CI)		Predictor	# Models	Selection probability	Estimate (Unconditional 95%CI)
Land mosaic occupancy	0.0599	H1	256	0.6610	0.136 (−0.104, 0.377)	0.0606	H1	256	0.9103	**0.205 (0.038, 0.371)**
		H2	256	0.6129	0.109 (− 0.105, 0.324)		H2	256	0.3885	0.036 (−0.069, 0.140)
		H3	256	0.2769	−0.031 (−0.144, 0.081)		H3	256	0.6448	−0.083 (−0.237, 0.071)
		H3^2^	256	0.2007	0.002 (−0.031, 0.034)		H3^2^	256	0.2670	−0.009 (−0.055, 0.037)
		M1	256	0.5897	0.106 (−0.110, 0.323)		M1	256	0.3218	0.031 (−0.071, 0.132)
		M2	256	0.2076	0.009 (−0.040, 0.058)		M2	256	0.1875	−0.003 (−0.038, 0.032)
		M3	256	0.2119	−0.023 (−0.125, 0.080)		M3	256	0.2046	0.009 (−0.038, 0.056)
		M3^2^	256	0.2131	−0.027 (−0.162, 0.109)		M3^2^	256	0.2668	−0.016 (−0.074, 0.043)
		ATC	256	0.4315	0.829 (−1.418, 3.075)		ATC	256	0.2035	0.069 (−0.336, 0.474)
Patch occupancy rate	0.2792	H1	128	0.1824	0.009 (−0.036, 0.053)	0.0608	H1	128	0.7879	0.159 (−0.045, 0.364)
		H2	128	0.8524	**0.192 (0.004, 0.380)**		H2	128	0.2904	0.023 (−0.058, 0.104)
		H3	128	0.9310	−**0.230 (**−**0.410,** −**0.051)**		H3	128	0.6866	−0.100 (−0.268, 0.068)
		H3^2^	128	0.1694	−0.000 (−0.019, 0.019)		H3^2^	128	0.2680	−0.004 (−0.046, 0.037)
		M1	128	0.9930	**0.263 (0.138, 0.388)**		M1	128	0.6371	0.109 (−0.093, 0.312)
		M2	128	0.1755	−0.005 (−0.036, 0.026)		M2	128	0.2285	0.015 (−0.053, 0.084)
		M3	128	0.2519	−0.031 (−0.146, 0.085)		M3	128	0.2265	0.015 (−0.050, 0.079)
		M3^2^	128	0.1715	−0.003 (−0.068, 0.062)		M3^2^	128	0.3286	−0.027 (−0.114, 0.061)
Extent of occupancy	0.3003	H1	32	0.7158	0.176 (−0.088, 0.440)	0.5950	H1	32	1,0000	**0.531 (0.429, 0.634)**
		H2	32	0.7216	0.223 (−0.106, 0.552)		H2	32	1,0000	**0.448 (0.358, 0.538)**
		H3	32	0.1404	−0.009 (−0.054, 0.035)		H3	32	0.9500	−**0.215 (**−**0.338,** −**0.091)**
		M1	32	0.6292	0.096 (−0.079, 0.270)		M1	32	0.1249	0.003 (−0.014, 0.020)
		M2	32	0.1997	0.016 (−0.044, 0.075)		M2	32	0.1947	0.012 (−0.033, 0.056)
		M3	32	0.2157	−0.035 (−0.162, 0.092)		M3	32	0.1100	0.002 (−0.013, 0.017)

The table provides Akaike weights of the best fitting models (wi) for each response variable, the number of models including each predictor, the selection probabilities, and model averaged regression coefficients with 95% confidence intervals. Predictors included in the best models are underlined. Coefficient estimates whose 95%CI excluded 0 are presented in bold.

## Discussion

This study examined the value of mosaic-level approaches to understand responses of patchy populations to land use change, with data gathered on water voles inhabiting land mosaics across a gradient of agricultural intensification. We found that mosaic-level attributes of water vole populations (land mosaic occupancy, rate of patch occupancy and extent of occupancy) were strongly related to the characteristics of patch networks (total habitat availability, and average patch size and isolation) and, to a lesser extent, the composition of the surrounding matrix. As the relationships uncovered derived from replicated sampling of land mosaics spanning a large variety of patch network and matrix characteristics, it is expected that they may provide general guidelines to address the conservation management of water voles over a wide range of landscape types. Overall, results were consistent with previous network-level field studies of metapopulations [21]–[24], and suggest that mosaic-level inference may provide useful empirical information for designing landscape mosaics favouring the persistence of patchy populations in fragmented landscapes.

### Mosaic-level effects on water voles

Water vole occupancy of land mosaics was primarily influenced by the total amount of habitat available, including the correlated effects of total habitat area and number of habitat patches. These results are consistent with other empirical studies examining the factors affecting metapopulation persistence in patch networks [21]–[24], and with mosaic-level studies revealing a dominant effect of habitat amount on species occupancy of landscape mosaics [6], [7], [61], [62]. Our results thus suggest that the regional distribution of water voles may be determined to a large extent by the availability of suitable habitats, irrespective of the composition and configuration of patch networks and the surrounding matrix. Reasons for the stronger effect observed during the wet season are uncertain, but they may be related to the higher mobility of individuals during this season [34], which may favour the colonization of land mosaics transitorily vacant during the dry season. Other studies have suggested that occupancy of a patch network may be influenced by dispersal from neighbouring networks [23], though further research is needed to confirm this view in our system.

Variation in the proportion of patches occupied by water voles across land mosaics appeared to be driven primarily by average patch area and isolation. This result is consistent with metapopulation theory [14]–[15], and with a few empirical studies examining the effects of patch attributes at the level of patch networks [21]–[24]. The positive effect of average patch area on patch occupancy rate was probably due to the lower risk of population extinction in larger patches, whereas the negative effect of patch isolation was probably related to the decline in the colonization probability of empty patches with increasing distance from occupied patches [14]–[15]. Reasons for the stronger effects observed during the dry season are uncertain, but they may be related to harsher environmental conditions during dry and hot Mediterranean summer. Habitat quality deteriorates and breeding activity strongly declines during the dry season [27], [29], [33], [34], which may increase the extinction risk of local populations, particularly in small patches inhabited by just a few individuals. Colonization probability of distant empty patches may also be reduced during the dry season, both because dispersal movements among patches may be particularly costly during hot and dry periods, and because reduced breeding output limits the abundance of potential dispersers [34]. It is thus possible that the patterns observed were a consequence of colonization-extinction dynamics of local populations like in classical metapopulations, though it could not be ruled out the possibility of individuals retreating from dryer areas in the dry season, but returning in the wet season. This is unlikely, however, due to the reduced lifespan of individuals (around 3–5 months [34]), and because extensive radio tracking in the study area never documented movement of adults between habitat patches [27].

The extent of occupancy was unrelated to patch-network features during the dry season, but during the wet season it was positively related to the amount of habitat and average patch area, and negatively so with average patch isolation. As individuals are reproducing during the wet season, they may be able to occupy rapidly the entire habitat available, which may justify the positive effects of total habitat amount and average patch size. However, average isolation may slow down the occupation of the entire habitat available within the patch network, thereby underlying the negative relationships observed with this variable. In contrast, during the dry season there is little breeding and the population is declining, and so the extent of occupancy may be more related to other limiting factors such as fine-scale habitat quality, or predation, which may influence the depletion of individuals at local patches (e.g., [63]).

The characteristics of the matrix had relatively little influence on water voles, which is in line with previous studies of the species [35]. The only consistent effect was the positive relationship between patch occupancy rate and irrigated agriculture, which was stronger during the dry season. It is possible that irrigation ditches and vegetated wet margins within the dry farmland facilitate movements of water voles, as also referred for *A. amphibius* populations in the UK, and other semi-aquatic mammals [39], [64]. Also, irrigation may favour the retention of wet conditions within some patches during the summer, which may improve habitat quality and thus reduce extinction probability. These two factors together may justify increases in the proportion of patches occupied in irrigated landscapes. Lack of additional matrix effects may indicate that dispersal of water voles through the agricultural matrix is little affected by land use type, with the possible exception of irrigated agriculture.

In terms of conservation, results suggest that water voles may be favoured in farmland landscape by maintaining land mosaics with a number of patches dominated by wet herbaceous vegetation, even if these are embedded in an intensive agricultural matrix. Achieving this goal requires the conservation of marginal herbaceous vegetation of temporary ponds, small streams and ditches, and areas with elevated water tables, which provide the habitat conditions needed by the species [27], [29], [32]–[34]. Water voles appeared relatively indifferent to the land use types surrounding habitat patches, which suggest that less attention may need to be given to the management of the matrix than what seems to be required by other Mediterranean voles of conservation concern [26]. Also in contrast to other Mediterranean farmland species (e.g., [28], [65]), water voles appeared to cope well with the conversion from traditional dry agriculture to modern irrigated crop and pasture production, probably because this may favour the retention of wet habitat patches and enhance dispersal probability during the dry summer conditions. There was some evidence, however, that irrigated agriculture may result in smaller habitat patches, which in the long run may conduct to lower persistence probability.

### Applications and limitations of mosaic-level approaches

Conservation planning in fragmented landscapes requires the identification of the attributes of habitat networks and the surrounding matrix that maximise the persistence of patchy populations of conservation concern [18]. This problem has been addressed primarily by estimating factors affecting population occurrence and abundance at the patch level [3], [4], and then scaling up to entire patch networks using analytic or simulation models [19], [20]. However, results of the present study and of a few metapopulation studies conducted at the network level [21]–[24], support the view that useful additional insights could be gained through empirical studies conducted at broad, mosaic-level approaches [6]. Specifically, mosaic-level approaches may be useful to provide empirical testing of some metapopulation model predictions, as well as to generate novel ideas that can be amenable to theoretical treatment or to be used in practical conservation management [21]–[24].

The key aspect of the mosaic-level approach as applied to patchy populations is that it involves replication of habitat networks, potentially encompassing a wide range of variation in composition and configuration attributes of patch networks and the surrounding matrix [6], [14]. As a consequence, this approach permits direct empirical estimates of the attributes of patch networks (and the surrounding matrix) that explain whether they support a patchy population or not, and as well as variation in the prevalence and abundance of the population across networks (e.g., [23], [61], [62], this study). In contrast, focal patch studies use patches as the units of replication, and thus their inference is made on factors influencing patterns of local occupancy or abundance (e.g., [16], [27]). Although network-level attributes can also be considered in focal patch approaches, their influence is measured in terms of their contribution to explain variation in occupancy or abundance at the level of individual patches. Therefore, the type of inferences that can be drawn from the mosaic-level approach is distinct from that obtained through focal patch studies, and so it may contribute with additional insights for the conservation management of patchy population.

Despite their potential value, mosaic-level approaches may also have some limitations, and may not be applicable to all types of patchy populations. One potential problem is that these studies require a number of patch networks with different sizes and configurations, which may either be unavailable, or to be too costly or time-consuming for sampling. This probably explains why these studies have remained relatively scarce, and have focused primarily on invertebrates occupying relatively small habitat networks, with most examples being based on relatively small sample sizes [21]–[25]. Limitations of sample sizes may reduce the strength of inferences that can be drawn from mosaic-level studies, though this should only be a shortcoming where a sufficiently large number of patch networks is unavailable for sampling. Another problem is that mosaic-level approaches may be too coarse for detecting fine scale processes affecting patchy populations, which may limit their utility for developing management guidelines for specific landscapes [66]. For instance, details of habitat patch quality may be blurred in land mosaic approaches, though this may be an important factor affecting metapopulation persistence [67], [68]. The geographic position of specific patches within a network may also influence metapopulation persistence, but this is typically not covered in mosaic-level studies [14], [15].

Considering their potential strengths and limitations, we suggest that the mosaic-level approach may be particularly suited for studies focusing on patchy populations occupying relatively small patch networks (e.g., invertebrates, small mammals), which can thus be sufficiently replicated at the regional scale (e.g., [21], [63]). However, as patchy populations may be affected by factors occurring at different spatial scales, it might be valuable to combine mosaic- and patch-level approaches in a single hierarchical framework, which to the best of our knowledge has been carried out only in a single study [24]. Finally, we suggest that the application of the mosaic level approach to several species and study systems might provide useful empirical evidence on the patch network and matrix attributes favouring the persistence of patchy populations in fragmented landscapes.

## Supporting Information

Figure S1 Variogram plot of the multiscale ordination (MSO) of redundancy analysis (RDA) relating patch-network and matrix gradients of land mosaics. The number of pairs of observations within each distance class is presented above the x-axis. The maximum extent for the interpretation of the variogram (vertical dashed line) is ca. 38 km. The residual variance shows no spatial correlation and the overall variogram is essentially flat, suggesting that patch-network and matrix relationships are scale-invariant (*p*-values of permutation tests for independence of residual variance always greater than 0.05, after Bonferroni correction for 9 simultaneous tests).
(TIF)

Figure S2 Scatterplots showing linear and quadratic relations of water vole response variables with patch-network and matrix characteristics of land mosaics. In each case, the best fitting curve (in red) was carried forward to multivariate analysis, based on Akaike weights (wi).
(TIF)
