# Supplementary figures and images for: Influence of Land Mosaic Composition and Structure on Patchy Populations: The Case of the Water Vole (Arvicola sapidus) in Mediterranean Farmland
Source: PLoS One. 2013 Jul 16;8(7):e69976. doi: 10.1371/journal.pone.0069976 (PMC3713055; doi:10.1371/journal.pone.0069976)

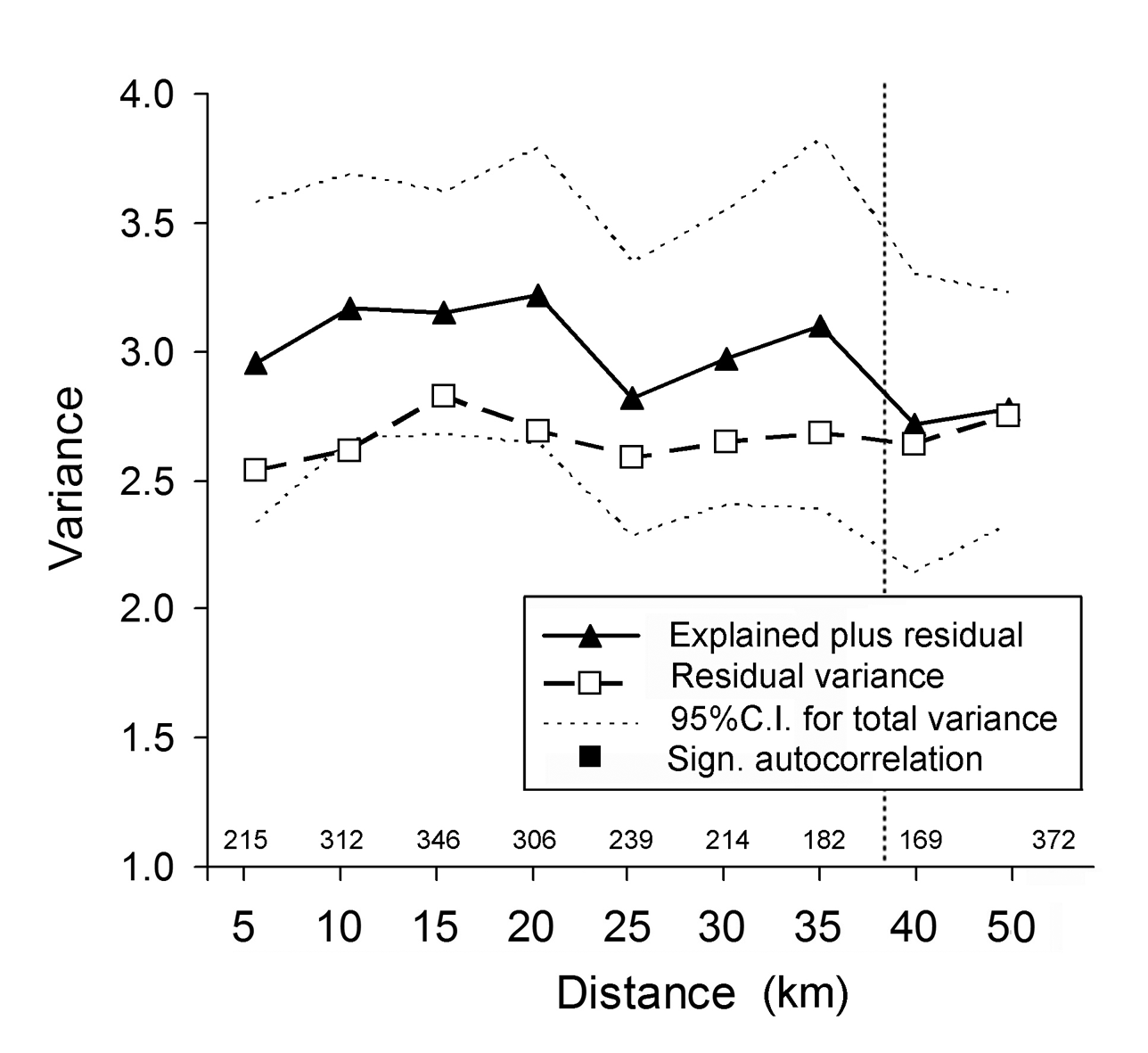

Supplement: Figure S1 — Variogram plot of the multiscale ordination (MSO) of redundancy analysis (RDA) relating patch-network and matrix gradients of land mosaics. The number of pairs of observations within each distance class is presented above the x-axis. The maximum extent for the interpretation of the variogram (vertical dashed line) is ca. 38 km. The residual variance shows no spatial correlation and the overall variogram is essentially flat, suggesting that patch-network and matrix relationships are scale-invariant (p-values of permutation tests for independence of residual variance always greater than 0.05, after Bonferroni correction for 9 simultaneous tests). (TIF) [file pone.0069976.s001.tif]

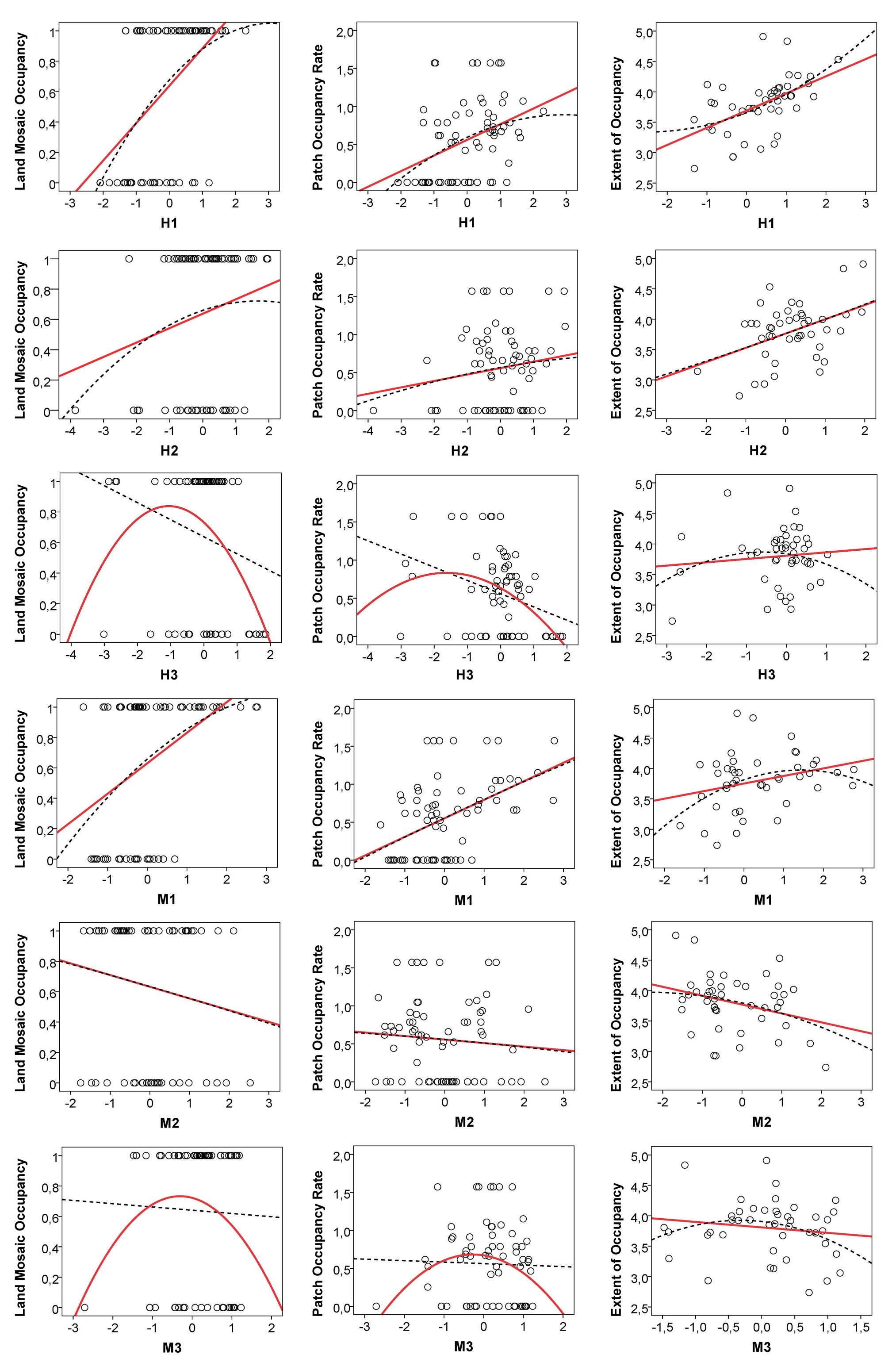

Supplement: Figure S2 — Scatterplots showing linear and quadratic relations of water vole response variables with patch-network and matrix characteristics of land mosaics. In each case, the best fitting curve (in red) was carried forward to multivariate analysis, based on Akaike weights (wi). (TIF) [file pone.0069976.s002.tif]
